# Retirement and mental health: dose social participation mitigate the association? A fixed-effects longitudinal analysis

**DOI:** 10.1186/s12889-017-4427-0

**Published:** 2017-05-30

**Authors:** Koichiro Shiba, Naoki Kondo, Katsunori Kondo, Ichiro Kawachi

**Affiliations:** 1 Departments of Health and Social Behavior/Health Education and Health Sociology, School of Public Health, The University of Tokyo, 7-3-1 Hongo, Bunkyo-ku, Tokyo, 113-0033 Japan; 2 Center for Preventive Medical Sciences, Chiba University, 1-8-1, Inohana, Chuo-ku, Chiba 260-8670 Japan; 3 Department of Gerontological Evaluation, Center for Gerontology and Social Science, National Center for Geriatrics and Gerontology, 7-430 Morikoka-cho, Obu-shi, Aichi 474-8511 Japan; 4 Department of Social and Behavioral Sciences, Harvard T.H. Chan School of Public Health, 677 Huntington Ave., 7th floor, Boston, MA 02115 USA

**Keywords:** Retirement, Mental health, Social participation, Fixed-effects, Japan, Older adults

## Abstract

**Background:**

Empirical evidence investigating heterogeneous impact of retirement on mental health depending on social backgrounds is lacking, especially among older adults.

**Methods:**

We examined the impact of changes in working status on changes in mental health using Japanese community-dwelling adults aged ≥65 years participating in the Japan Gerontological Evaluation Study between 2010 and 2013 (*N* = 62,438). Between-waves changes in working status (“Kept working”, “Retired”, “Started work”, or “Continuously retired”) were used to predict changes in depressive symptoms measured by the Geriatric Depression Scale. First-difference regression models were stratified by gender, controlling for changes in time-varying confounding actors including equivalised household income, marital status, instrumental activities of daily living, incidence of serious illnesses and family caregiving. We then examined the interactions between changes in working status and occupational class, changes in marital status, and post-retirement social participation.

**Results:**

Participants who transitioned to retirement reported significantly increased depressive symptoms (β = 0.33, 95% CI: 0.21–0.45 for men, and β = 0.29, 95% CI: 0.13–0.45 for women) compared to those who kept working. Men who were continuously retired reported increased depressive symptoms (β = 0.13, 95% CI: 0.05–0.20), whereas males who started work reported decreased depressive symptoms (β = −0.20, 95% CI: -0.38–-0.02). Men from lower occupational class (compared to men from higher class) reported more increase in depressive symptoms when continuously retired (β = −0.16, 95% CI: -0.25–-0.08). Those reporting recreational social participation after retirement appeared to be less influenced by transition to retirement.

**Conclusions:**

Retirement may increase depressive symptoms among Japanese older adults, particularly men from lower occupational class backgrounds. Encouraging recreational social participation may mitigate the adverse effects of retirement on mental health of Japanese older men.

**Electronic supplementary material:**

The online version of this article (doi:10.1186/s12889-017-4427-0) contains supplementary material, which is available to authorized users.

## Background

Population aging is occurring worldwide and an increasing number of older adults are engaging in work in late life. Japan is experiencing the fastest rate of population ageing in the world. In Japan, the labor force participation rate among people aged 65 years or older was 21.3% in 2014, which ranked 7th among all OECD countries [1], and the population of older workers increased from 4.8 million in 2004 to 6.8 million in 2014 [2]. To maintain both the quality of life of each older individual and the financial sustainability of healthcare and long-term care systems, preventing physical and mental impairments is the key goals of public health measures for the aged [3]. Specifically, the maintenance of mental health counts as it could be strongly affected by the stressful life events that are likely to happen in older ages. Among those life events, in this study, we focused on retirement, the major events that most people experience.

Transition from work to retirement is a major life event in most people’s lives. However, there is controversy about the impact of retirement on mental health [4]. While many studies done in European countries suggest that retirement is beneficial to mental health [5–8] or has no health impact [9–11], results from some studies in Asian populations indicate the opposite, i.e. the transition to retirement is detrimental to mental health [12, 13]. These geographic differences in the association between retirement and mental health may be partly due to the differences in employment conditions and how retirements are culturally perceived by people in each region.

There are several potential mechanisms through which retirement may have adverse impact on mental health. Above and beyond the immediate reduction in income, social role theory posits that retirement results in the loss of many non-financial benefits of work, such as opportunities for health-promoting social contacts and access to social support that can buffer the adverse effects of stress [14–16]. Activity theory also posits that the transition to retirement results in reduced wellbeing for individuals for whom work provided meaning in life [17].

Moreover, the impact of retirement on mental health may not be universal and could vary between individuals depending on his/her occupational class or social situation (e.g. the presence of social support in the home environment from a marital partner) [4, 6, 18, 19]. In addition, social participation after retirement may compensate for any adverse effect of retirement stemming from loss of sense of meaning in life, which has been suggested to be linked to mental health [4, 12, 13, 20]. However, the potential impact of other types of social participation such as hobby clubs remains unknown.

In the present study, we sought to evaluate [1] the association between changes in working status and changes in depressive symptoms in a cohort of community-dwelling Japanese older adults, [2] whether the association can be mediated by changes in social contacts and social support, [3] the interaction between changes in working status and occupational class, changes in marital status, and social participation after retirement.

## Methods

### Data

We used data from the 2010 and 2013 waves of Japan Gerontological Evaluation Study (JAGES) project. Detailed descriptions of JAGES are available elsewhere [21, 22]. In brief, the JAGES conducted postal surveys targeting physically and cognitively independent community-dwelling older adults aged ≥65 years in Japan. The baseline survey was conducted in 2010 and we sampled 112,123 older adults residing in 31 municipalities in Japan (Response rate = 66.3%). The second survey was conducted in 2013 in which we sampled 137,736 older adults residing in 30 municipalities in Japan (Response rate = 71.1%). Twenty-four municipalities participated in both years. Sixty-two thousand four hundred thirty-eight participants responded to both surveys.

The 2013 wave of JAGES dataset consisted of five modules. The question inquiring about participants’ sense of meaning in life was measured only in one of the five modules, which was only mailed to one-fifth of the total sample. We used respondents to this module (*N* = 12,487) to test the interaction between retirement and two types of social participation, as well as whether a sense of meaning in life mediated this relationship. All variables used in this study were self-reported. Ethical approval of this study was obtained from the ethics review board of the University of Tokyo Medical School.

### Measurement

#### Changes in depressive symptoms

Depressive symptoms were measured at both waves using the validated Japanese short version of the Geriatric Depression Scale (GDS-15) [23] Total scores could range from 0 to15, where higher scores indicate more depressive symptoms. GDS-15 has been commonly used as a screening tool for depression among older adults with a cutoff of 5 or above to indicate clinical depression [23, 24]. We used changes in total GDS-15 scores from 2010 to 2013 as the continuous outcome variable in this study.

#### Changes in working status

Information on working status (either “currently working” or “retired and not currently working”) was collected in both 2010 and 2013. Thus, no one among the subjects was considered to be partially retired. We categorized subjects as “Kept working (working/working)”, “Retired (working ➔ not working)”, “Started work (not working ➔ working)”, or “Continuously retired (not working ➔ not working)” based on patterns of changes in working status from 2010 to 2013.

#### Changes in social contacts and social support

We used two variables to tap changes in social contacts. First, we asked the number of friends and acquaintances the subjects met in the past month at both waves, and then, changes in the answers were modeled as a continuous variable. Second, we asked subjects “What is the relationship between you and the person you often meet?” At each wave, we focused on individuals who maintained social contacts with colleagues in the workplace. As for social support, we collected information at both waves on whether or not subjects received emotional (‘Do you have anyone who will listen to you when you have worries and complains?’) and instrumental (‘Do you have anyone who can take care of you when you are sick in bed for a few days?’) social support from their friends. Between-wave changes in perception of social supports from friends were made into a categorical variable.

#### Occupational class

The occupation that subjects had engaged in for the longest period of time was asked at both waves. Since it is unlikely that the longest job of older people aged 65 years or older change within 3 years (from 2010 to 2013), we treated this as a time-invariant variable. Professionals and managers were categorized as “higher occupational class”. Clerical support workers, service and sales workers, craft and related trades workers, skilled agricultural, forestry, and fishery workers, and others were categorized as “lower occupational class”.

#### Social participation/a sense of meaning in life

Participation in different clubs/groups was asked at both waves. We defined “social participation with roles” as participation in volunteering clubs or neighborhood councils. We defined “recreational social participation” as participation in sports organizations, hobby clubs, or older adults clubs. A sense of meaning in life was measured in 2013 by asking “Do you feel a sense of meaning in life” (yes/no).

#### Covariates

Changes in equivalised household income, marital status, instrumental activities of daily living, incidence of serious illnesses and family caregiving were treated as time-varying covariates. These variables are potential confounding factors, which could have influence on both retirement decision and mental health. Household income at each wave was equivalised in order to adjust for the number of members within households. Marital status of subjects at each wave was defined either “married” or “non-married (including being widowed, divorced, or single)”. Subjects’ instrumental activities of daily living (IADL) were measured using the Tokyo Metropolitan Institute of Gerontology (TMIG) Index of Competence as a broad indicator of physical health. TMIG Index of Competence was validated using the data of Japanese older community residents [25] and its total scores could range from 0 to13, where higher scores indicates more independency in daily livings. Incidence of serious illnesses and need for caregiving was assessed in 2013 by administering a checklist for events occurring in the past year.

### Statistical analysis

As for missing values in some variables used in the analysis, we executed multiple imputation by chained equation method to create 100 data sets without missing value. We used the FCS statement of the MI procedure in SAS version 9.3 to create imputed data sets. Then, we excluded those who indicated depression in 2010 (GDS score of 5 or more as described below), those who never worked, and those who answered inconsistently to the question about the occupational class at the two time points. We ran all models using each data set and, using MIANALYZE procedure, we obtained the final estimates combining 100 estimates derived from 100 analyses.

We used first-difference models stratified by gender to investigate the main effects of changes in working status on changes in depressive symptoms with full-sample data (**Model 1**). The first-difference models tool the changes in all variables in the models, which enable to can control for all observed and unobserved time-invariant confounders (e.g. age and education) by cancelling out the estimates of time-invariant confounders. In model 2, we added variables regarding changes in social contacts and social support to test whether these changes could explain the association between changes in working status and changes in depressive symptoms. In model 3, we added an interaction term between changes in working status and occupational class. In model 4, we added an interaction term between transition to retirement and changes in marital status.

In the analysis involving a sense of meaning in life, we analyzed only one of the five sub-samples of JAGES data because it is the only module that contains information on participants’ sense of purpose in life. In model 5**,** we used the first-difference model again to test the main effect of changes in working status on depressive symptoms among this sub-population. In the model 6, we added the interaction term between transition to retirement and two types of social participation. Lastly, in model 7, we added sense of meaning in life as a covariate to check whether it mediated the interaction between retirement and social participation.

We stratified data by gender instead of modeling gender interaction effects to make it easy to interpret our results and prevent the problems of multicollinearity on interaction terms.

## Results

Descriptive analysis showed that subjects in the full-sample dataset and the sub-sample dataset were broadly similar in terms of their background characteristics (Table 1). Nearly a quarter of male and female subjects indicated depressive symptoms at baseline.

**Table 1:** Demographic Characteristics of Subjects from Full-dataset and Sub-dataset by Gender

	Full-sample					Sub-sample				
	Men		Women			Men	Women			
	*N* = 28,868		*N* = 33,569			*N* = 5866	*N* = 6621			
	Mean	SD	Mean	SD	*p*-value	Mean	SD	Mean	SD	*p*-value
Age in 2010	72.9	5.6	73.1	5.6	<.0001	72.9	5.6	73.1	5.6	0.02
GDS-15 score change (2010 → 2013)	0.1	2.4	0.03	2.4	0.0005	0.1	2.5	−0.01	2.3	0.02
	n	%	n	%		n	%	n	%	
Working status in 2010										
Working status	8095	30.3	5287	19.2	<.0001	1687	31.1	1058	19.5	<.0001
Not working	17,530	65.6	16,556	60		3510	64.7	3231	59.6	
Never worked	1087	4.1	5730	20.8		232	4.3	1133	20.9	
Missing	2156		5996			437		1199		
Depression in 2010										
Having depression in 2010	6353	25.5	6816	25.5	0.84	1340	26.4	1354	25.7	0.4
Not having depression in 2010	18,602	74.5	19,878	74.5		3730	73.6	3913	74.3	
Missing	3913		6875			796		1354		
Household income in 2010 (10,000 Japanese Yen)										
< 300	12,129	45.6	15,051	54	<.0001	2429	44.8	2921	53.2	<.0001
300–600	9759	36.7	8427	30.2		2027	37.4	1676	30.5	
< 600	4711	17.7	4393	15.8		969	17.9	897	16.3	
Missing	2269		5698			441		1127		
Marital status in 2010										
Married	24,608	87.9	19,790	61.3	<.0001	5007	88	3894	61.1	<.0001
Non-married	3374	12.1	12,520	38.8		685	12	2484	39	
Missing	886		1259			174		243		
IADL in 2010										
13	9401	36.6	15,590	53.1	<.0001	1882	36	3052	52.7	<.0001
10, 12	12,959	50.4	11,548	39.3		2642	50.5	2313	40	
≤ 9	3351	13	2250	7.7		710	13.6	423	7.3	
Missing	3157		4181			632		833		
The number of friends subjects met in the past 1 month in 2010										
0	2041	7.6	1065	3.5	<.0001	419	7.7	198	3.3	<.0001
1, 5	11,426	42.5	12,589	40.8		2302	42.3	2495	41.1	
6-	13,396	49.9	17,236	55.8		2728	50.1	3385	55.7	
Missing	2005		2679			417		543		
Colleague as a friend who subjects met frequently in 2010										
Having colleague as a friend	11,439	42.4	8027	25.4	<.0001	2365	43.2	1522	24.4	<.0001
Not having colleague as a friend	15,563	57.6	23,625	74.6		3116	56.9	4713	75.6	
Missing	1866		1917			385		386		
Emotional social support from friends in 2010										
Having emotional social support	7233	26.6	15,156	47.9	<.0001	1425	25.7	2974	47.9	<.0001
Not having emotional social support	19,950	73.4	16,482	52.1		4112	74.3	3234	52.1	
Missing	1685		1931			329		413		
Instrumental social support from friends in 2010										
Having instrumental social support	632	2.3	2144	6.8	<.0001	114	2.1	400	6.4	<.0001
Not having instrumental social support	26,793	97.7	29,539	93.2		5454	97.8	5844	93.6	
Missing	1444		1886			298		377		
Occupational class										
Higher	9265	36.2	3031	12.6	<.0001	1879	36.5	605	12.9	<.0001
Lower	16,325	63.8	20,940	87.4		3268	63.5	4083	87.1	
missing	3153		6394			698		1326		
Social participation with roles in 2013										
Participated in social activities with roles	6536	26.3	7361	27.8	0.001	1292	25.6	1461	28	0.005
Not participated in social activities with roles	18,346	73.7	19,132	72.2		3764	74.5	3754	72	
missing	3986		7076			810		1406		
Recreational social participation in 2013										
Participated in recreational social activities	11,714	46.5	16,067	58	<.0001	2367	46.3	3167	57.9	<.0001
Not participated in recreational social activities	13,458	53.5	11,657	42.1		2751	53.8	2304	42.1	
missing	3696		5845			748		1150		
A sense of meaning in life in 2013										
Having a sense of meaning in life in 2013						4666	86.2	5036	84.2	0.003
Not having a sense of meaning in life in 2013						748	13.8	945	15.8	
missing						452		640		

*GDS-15* the short version of the Geriatric Depression Scale (ranging from 0 to 15, higher score indicates more depressive symptoms). Depression in 2010 was defined by GDS-15 score of 5 or above. Non-married includes being divorced, widowed, and single. *IADL* Instrumental activities of daily living (ranging from 0 to 13, higher score indicates more independency in daily livings). Higher occupational class = professionals and engineering, managers. Lower occupational class = clerical support workers, service and sales workers, craft and related trades workers, skilled agricultural, forestry, and fishery workers, others. Social participation with roles = volunteering clubs, neighborhood councils. Recreational social participation = sports organizations, hobby clubs, or older adults clubs. Full-sample: subjects from all of the five modules of JAGES. Sub-sample: subjects from only one of the five modules from JAGES that includes information on a sense of meaning in life. We used t-test for age and changes in GDS score and chi-square test for other variables to calculate *p*-values

For men, we observed significant increases in GDS score among those who transitioned to retirement (β = 0.33, 95% CI: 0.21–0.45) and those who were continuously retired (β = 0.13, 95% CI: 0.05–0.20) compared to those who kept working (**Model 1** in Table 2). For women, we observed significant increases in GDS score among those who transitioned to retirement (β = 0.29, 95% CI: 0.13–0.45) compared to those who kept working. After including changes in social contacts and social support in **Model 2**, significant associations observed in **Model 1** were overall unchanged and remained statistically significant.

**Table 2:** Multiple linear regression of Changes in GDS score on change in working status by gender (full-sample)

Independent variables	Dependent Variable: Changes in GDS-15 score from 2010 to 2013							
	Model 1				Model 2			
	Men		Women		Men		Women	
	β coefficient (95%CI)							
Changes in working status (2010–2013)								
Kept working	Ref.	Ref.	Ref.	Ref.	Ref.	Ref.	Ref.	Ref.
Retired	0.33	(0.21, 0.45)	0.29	(0.13,0.45)	0.33	(0.21,0.45)	0.28	(0.12,0.44)
Started work	−0.20	(−0.38, −0.02)	0.10	(−0.13,0.33)	−0.21	(−0.39,-0.03)	0.10	(−0.13,0.33)
Continuously retired	0.13	(0.05, 0.20)	0.05	(−0.04,0.14)	0.10	(0.02,0.18)	0.05	(−0.03,0.14)
Changes in Equivalized household income (10,000 JPY)	−0.0004	(−0.0007, −0.0002)	−0.0005	(−0.0007,-0.0002)	−0.0004	(−0.0007,-0.0002)	−0.0004	(−0.0007,-0.0002)
Changes in IADL	−0.24	(−0.26, −0.21)	−0.28	(−0.31,-0.25)	−0.23	(−0.25,-0.2)	−0.26	(−0.3,-0.23)
Incidence of stressful life events in the past 1 year								
Serious illnesses	0.59	(0.47, 0.70)	0.70	(0.54,0.87)	0.58	(0.46,0.7)	0.70	(0.53,0.87)
Started family caregiving	0.54	(0.35, 0.73)	0.55	(0.38,0.72)	0.54	(0.34,0.73)	0.55	(0.38,0.72)
Changes in marital status								
Married – Married	Ref.	Ref.	Ref.	Ref.	Ref.	Ref.	Ref.	Ref.
Married - Not married	0.48	(0.26, 0.70)	0.15	(−0.02,0.32)	0.48	(0.26,0.7)	0.16	(−0.01,0.33)
Not married – Married	−0.04	(−0.45, 0.36)	0.54	(0.13,0.95)	−0.04	(−0.44,0.37)	0.51	(0.11,0.92)
Not married - Not married	0.20	(0.08,0.31)	0.11	(0.04,0.18)	0.21	(0.09,0.33)	0.11	(0.04,0.18)
The number of friends subjects met in the past 1 month					−0.02	(−0.03,-0.01)	−0.03	(−0.04,-0.02)
Colleagues as a friend who subjects met frequently								
Continuously having colleagues as a friend					Ref.	Ref.	Ref.	Ref.
Lost colleagues as a friend					0.01	(−0.09,0.11)	0.06	(−0.06,0.19)
Continuously not having colleagues as a friend					0.08	(0.004,0.16)	−0.003	(−0.09,0.09)
Newly had colleagues as a friend					0.04	(−0.08,0.15)	−0.03	(−0.17,0.1)
Emotional support from friends								
Continuously having emotinal social support from friends					Ref.	Ref.	Ref.	Ref.
Lost emotional social support from friends					0.16	(0.04,0.28)	0.16	(0.04,0.27)
Continuously not having emotional social support from friends					0.21	(0.12,0.3)	0.12	(0.04,0.2)
Newly had emotional support from friends					0.08	(−0.03,0.19)	−0.01	(−0.12,0.1)
Instrumental support from friends								
Continuously having instrumental social support from friends					Ref.	Ref.	Ref.	Ref.
Lost instrumental social support from friends					0.23	(−0.2,0.66)	0.19	(−0.05,0.42)
Continuously not having instrumental social support from friends					0.05	(−0.32,0.42)	0.06	(−0.13,0.25)
Newly had instrumental support from friends					−0.01	(−0.42,0.41)	−0.08	(−0.33,0.16)

*GDS-15* the short version of the Geriatric Depression Scale (ranging from 0 to 15, higher score indicates more depressive symptoms). Subjects are those who did not show depression at baseline (GDS score < 5). *IADL* Instrumental activities of daily living (ranging from 0 to 13, higher score indicates more independency in daily livings). Non-married includes being divorced, widowed, and single. Data of full-sample (all of the 5 sub-versions of JAGES datasets) was used for these analysis

**Fig. 1:**
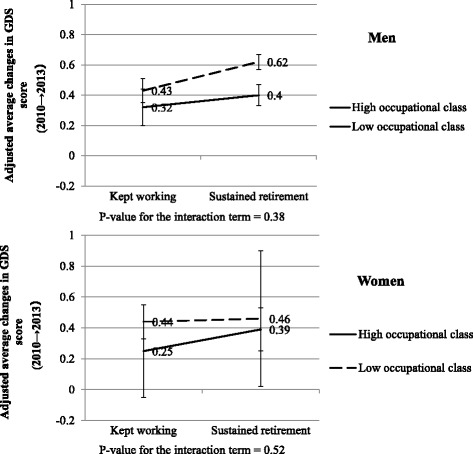
Adjusted average changes in GDS score by changes in working status and occupational class. GDS: the short version of the geriatric depression scale (ranging from 0 to 15, higher score indicates more depressive symptoms). Adjusted for changes in time varying confounding factors including equivalised houshold income, IADL limitation, marital status, stressful life events, social relationships. Subjects are those who did not show depression at baseline (GDS score < 5). Occupational status: 1 = high (professionals and engineering, managers), 0 = low (clerical support workers, service and sales workers, craft and related trades workers, skilled agricultural, forestry, and fishery workers, others)

When including interaction terms (Additional file 1 **:** Table S1), we did not find any statistically clear evidence on that the association between changes in working status and GDS scores were altered by occupational class (**Model 3**), though there were tendency that the relative increase in GDS score among men who were continuously retired were smaller among high occupational classes than lower occupational class (β = −0.08, 95% CI: -0.22–0.07) (Fig. 1). There was no significant interaction between changes in working status and occupational class among women. We observed no significant interactions between transition to retirement and changes in marital status among men and women (**Model 4**).

Analyses using the sub-dataset showed consistent results: significant increases in GDS score among men who transitioned to retirement (β = 0.40, 95% CI: 0.12–0.68) as well as men who were continuously retired (β = 0.29, 95% CI: 0.11–0.47) compared to men who kept working (**Model 5** in Additional file 2: Table S2). Women who transitioned to retirement reported marginally significant increases in GDS scores (β = 0.32, 95% CI: -0.04–0.68, *p* = 0.09).

**Fig. 2:**
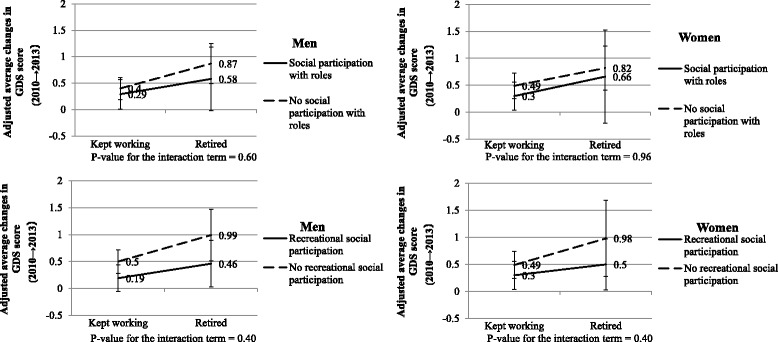
Adjusted average changes in GDS score by changes in working status and post-retirement social participation. GDS: the short version of the geriatric depression scale (ranging from 0 to 15, higher score indicates more depressive symptoms). Adjusted for changes in time varying confounding factors including equivalised houshold income, IADL limitation, marital status, stressful life events, social relationships. Subjects are those who did not show depression at baseline (GDS score < 5). Social participation with roles includes volunteering clubs and neighborhood councils. Recreational social participation includes sports organizations, hobby clubs, and older adults clubs

Although statistical evidence was weak but there was a trend among men and women that increase in depressive symptoms was less among those who participated in recreational social participation (β = −0.22, 95% CI: -0.72–0.29 for men, and β = −0.28, 95% CI: -0.92–0.37 for women) (**Model 6** in Additional file 3: Table S3, Fig. 2 **)**. After additional adjustment for whether or not subjects have a sense of meaning in life (**Model 7** in Additional file 3: Table S3), the magnitude of interaction effect between transition to retirement and recreational social participation among men considerably reduced (β = −0.05, 95% CI: -0.54–0.44), whereas it did not change among women (β = −0.27, 95% CI: -0.89–0.35). Notably, having a sense of meaning in life was strongly associated with increased depressive symptoms among men and women (β = −2.66, 95% CI: -3.00–-2.32 for men, and β = −1.84, 95% CI: -2.11-1.57 for women).

## Discussion

We summarize our key findings. First, the transition to retirement in both men and women and sustained retirement in men were associated with increased depressive symptoms among Japanese older adults. Second, the associations between retirement and increased depressive symptoms remained statistically significant after additionally adjusting for changes in social contacts and social support, suggesting that they did not mediate the impact of retirement on mental health at least among Japanese older adults. Third, there was a statistically non-significant trend that men from higher occupational class backgrounds appeared to be less influenced by sustained retirement, whereas we did not find such a trend among women. Fourth, we found no interaction between changes in working status and changes in marital status. Finally, there was a statistically non-significant trend that men and women who reported recreational social participation after retirement appeared to be less adversely impacted by retirement. Importantly, the magnitude of interaction effect between transition to retirement and post-retirement recreational social participation substantially reduced only among men after additionally adjusting for a sense of meaning in life. This, if causal, may suggest that having a sense of meaning in life through social participation may be crucial in mitigating the adverse impact of retirement on mental health – particularly for Japanese men.

In our data, nearly a quarter of male and female subjects indicated depressive symptoms at baseline defined by the GDS score of 5 or more. Another population-based study in Japan targeting people over the age of 64 reported that prevalence of those who scored more than 5 in GDS-15 was 23.8%, which is compatible to our data [26].

Our finding that retirement may increase depressive symptoms is inconsistent with many studies conducted in European countries [5–11], whereas a previous study in another Asian population supports our result [12, 13]. We speculate that, for Japanese workers (men especially), the loss of non-financial benefits of work outweighs the other benefits of retirement such as a relief from work-related stress overall. Interestingly, we found that changes in social contacts and social supports failed to explain the association between retirement and depressive symptoms. Although convincing evidence is lacking, the adverse effect of retirement may be attributable to a loss of other benefits of work including sense of meaning in life. Indeed, the literature suggests that many Japanese workers tend to find their purpose in life from work [27]. Retirement among Japanese workers may thus result in a loss of a sense of meaning in life.

Among Japanese workers who experienced the post-War period of rapid economic growth, employment was characterized by long hours of work as well as a shared sense of sacrifice with the goal of rebuilding the nation in the aftermath of the destruction wrought by the Second World War [28]. The participants in JAGES study -- all of whom were aged over 65 years at baseline in 2010 -- belong to this cohort. In return for their sacrifice of leisure and family life, Japanese workers of this generation were rewarded with a life-time employment guarantee. Commitment to the company thereby molded the identity of an entire generation of Japanese workers. It is in this context that we may interpret the findings of the present study. The transition to retirement for many Japanese workers entails the severing of links with work colleagues as well as loss of a sense of meaning in life. It is important to note at the same time, however, that the post-War lifetime employment guarantee has broken down in Japanese society in the aftermath of the 1991 economic bubble collapse. Our findings may therefore not apply to future generations of Japanese workers.

Our results also suggest that retirement may be more toxic among Japanese men from lower occupational class backgrounds compared to managerial/professional occupations. One explanation is that men from high SES backgrounds may have extra social roles besides work-related social roles, and therefore, may be less affected by a loss of work-related role due to retirement. Additionally, there may be some health benefits of retirement for the managerial/professional class. Indeed, mortality trends in Japan by occupational class indicate that suicide rates in these groups have risen sharply in the past two decades, possibly as a result of the pressures stemming from economic stagnation [29, 30]. It is possible that retirement can be somewhat beneficial for those workers as it relieves them of the work-related distress. The observed wide confidence intervals for the interaction terms between changes in working status and occupational class among women may be due to the small number of women who were from high-occupational background. Indeed, among female JAGES subjects in 2010, only 12.6% were categorized as high occupational class.

Our findings also indicate the trend that participation in recreational activity could mitigate the increase in depressive symptoms among Japanese retired men and women but perhaps through different mechanisms. Results from the model additionally adjusting for a sense of meaning in life suggests that recreational social participation may benefit retired Japanese men by compensating for a loss of a sense of meaning in life due to retirement. However, this was not the case for retired women. We speculate that recreational social participation for these retired women had differential meaning such as increased opportunities for informal socializing, rather than provision of a sense of meaning in life. Indeed, Takagi et al. also argued that there was a gender difference in mechanisms through which social participation enhances mental health [24]. We found no significant interaction between transition to retirement and social participation with roles including volunteerism, which was inconsistent with preceding findings [12, 13]. Participation in activities involving roles may not be completely voluntary and some participants might have been feeling a psychological distress due to “role strain”, namely a pressure to accomplish their roles [20, 31]. This may explain the non-significant interaction effect of social participation with roles and its wide variance.

Ours is, to our knowledge, the first study to evaluate the association between changes in working status and changes in depressive symptoms using data of Japanese older people. Strengths of our study include the large sample size of older adults with repeated measures of both outcomes and exposures, and the use of first-difference models to account for all time-invariant confounding factors. Nonetheless, several limitations should be noted. First, there is a possibility of reverse causation, such that workers are more likely to seek retirement when they feel depressed [32]. Although we partly addressed this issue by excluding subjects who showed depression at baseline from the analyses, future studies should specify reasons for retirement to permit causal inference. Second, unmeasured confounding factors may also exist. Even though we controlled for all observed/unobserved time-invariant confounding factors and several possible time-variant confounding factors, there may be other stressful life events or circumstances, which causes changes in working status and changes in depressive symptoms. Third, some of our measurements were admittedly crude. We used changes in received social supports from “friends” as a measurement of changes in work-related social relationships. However, it is obvious that social relationships people obtain from work are not necessarily reported as “friends”. Fourth, we evaluated social participation at the same time with our final outcome. Since we hypothesized that post-retirement social participation could mitigate the possibly adverse impact of retirement on mental health, it is ideal to measure social participation before measuring post-retirement mental health. Fifth, generalizability of our findings is limited. As the effect of retirement on mental health is presumably culture dependent, our findings may not be generalizable to other countries or future generations. Lastly, our single item measurement of “a sense of meaning in life” is not validated, and therefore, it may not successfully capture a subject’s sense of meaning in life.

## Conclusions

Retirement may increase depressive symptoms among Japanese older people, possibly men with lower occupational class backgrounds in particular. An important public health implication of our study is that there is a possibility that encouraging recreational social participation may compensate for a loss of a sense of meaning in life due to retirement and mitigate the potentially adverse effects of retirement on mental health of Japanese older men.

## Additional files

Additional file 1: Table S1. Regression Coefficients of Changes in Working Status and Interactions with occupational class (Model 3) and changes in marital status (Model 4) by gender (full-sample). (DOCX 17 kb)

Additional file 2: Table S2. Multiple linear regression of Changes in GDS score on changes in working status by gender (sub-sample). (DOCX 17 kb)

Additional file 3: Table S3. Regression Coefficients of Changes in Working Status and Interactions with social participation by gender (sub-sample). (DOCX 14 kb)

## Abbreviations

GDS-15: Short version of the Geriatric Depression Scale
IADL: Instrumental activities of daily living
JAGES: Japan gerontological evaluation study
TMIG: Tokyo Metropolitan Institute of Gerontology
