# Supplementary material for: Retirement and mental health: dose social participation mitigate the association? A fixed-effects longitudinal analysis
Source: BMC Public Health. 2017 May 30;17:526. doi: 10.1186/s12889-017-4427-0 (PMC5450308; doi:10.1186/s12889-017-4427-0)
Supplement: Supplementary file 1 — Regression Coefficients of Changes in Working Status and Interactions with occupational class (Model 3) and changes in marital status (Model 4) by gender (full-sample). (DOCX 17 kb) [file 12889_2017_4427_MOESM1_ESM.docx]

**Table S1.** Regression Coefficients of Changes in Working Status and Interactions with occupational class (Model 3) and changes in marital status (Model 4) by gender (full-sample)

| Independent variables | Dependent Variable: Changes in GDS-15 score from 2010 to 2013 | | | | | | | | |
| --- | --- | --- | --- | --- | --- | --- | --- | --- | --- |
|  | Model 3 | | | | | Model 4 | | | |
|  | Men | | | Women | | Men | | Women | |
|  | β coefficient (95%CI) | | | | | | | | |
| **Changes in working status** |  |  |  | |  |  |  |  |  |
| Kept working | Ref. | Ref. | Ref. | | Ref. | Ref. | Ref. | Ref. | Ref. |
| Retired | 0.31 | (0.16, 0.45) | 0.14 | | (0.03, 0.26) | 0.33 | (0.21,0.46) | 0.27 | (0.07,0.47) |
| Started work | -0.05 | (-0.25, 0.16) | 0.05 | | (-0.06, 0.17) | -0.21 | (-0.39,-0.03) | 0.10 | (-0.13,0.33) |
| Continuously retired | 0.16 | (0.07, 0.25) | 0.002 | | (-0.09, 0.10) | 0.10 | (0.02,0.18) | 0.05 | (-0.03,0.14) |
| **Interaction terms** |  |  |  | |  |  |  |  |  |
| Kept working *High occupational class | Ref. | Ref. | Ref. | | Ref. |  |  |  |  |
| Retired * High occupational class | -0.02 | (-0.31, 0.27) | -0.08 | | (-0.60, 0.44) |  |  |  |  |
| Started work * High occupational class | 0.07 | (-0.21, 0.36) | 0.07 | | (-0.38, 0.52) |  |  |  |  |
| Continuously retired * High occupational class | -0.08 | (-0.22, 0.07) | 0.08 | | (-0.20, 0.36) |  |  |  |  |
| Retired* Married-Married |  |  |  | |  | Ref. | Ref. | Ref. | Ref. |
| Retired * Married - Not married |  |  |  | |  | -0.004 | (-0.66,0.66) | -0.24 | (-0.87,0.39) |
| Retired * Not married - Married |  |  |  | |  | 0.34 | (-0.98,1.65) | 0.11 | (-1.35,1.56) |
| Retired * Not married - Not married |  |  |  | |  | -0.10 | (-0.52,0.33) | 0.06 | (-0.25,0.37) |

Adjusted for changes in time varying confounding factors including equivalised houshold income, IADL limitation, marital status, stressful life events, social relationships. GDS-15: the short version of the Geriatric Depression Scale (ranging from 0 to 15, higher score indicates more depressive symptoms). Subjects are those who did not show depression at baseline (GDS score < 5). Occupational status:1=higher (professionals and engineering, managers) 0=lower (clerical support workers, service and sales workers, craft and related trades workers, skilled agricultural, forestry, and fishery workers, others). Changes in marital status: Reference = Married – Married, Non-married includes being divorced, widowed, and single. Main effects of occupational class and changes in marital status were included in each model. Data of full-sample (all of the 5 sub-versions of JAGES datasets) was used for these analyses.
