# Supplementary material for: Retirement and mental health: dose social participation mitigate the association? A fixed-effects longitudinal analysis
Source: BMC Public Health. 2017 May 30;17:526. doi: 10.1186/s12889-017-4427-0 (PMC5450308; doi:10.1186/s12889-017-4427-0)
Supplement: Supplementary file 2 — Multiple linear regression of Changes in GDS score on changes in working status by gender (sub-sample). (DOCX 17 kb) [file 12889_2017_4427_MOESM2_ESM.docx]

**Table S2.** Multiple linear regression of Changes in GDS score on changes in working status by gender (sub-sample)

| Independent variables | Dependent Variable: Changes in GDS score from 2010 to 2013 | | | |
| --- | --- | --- | --- | --- |
|  | Model 5 | | | |
|  | Men | | Women | |
|  | β coefficient (95% CI) | | | |
| **Changes in working status (2010 - 2013)** |  |  |  |  |
| Kept working | Ref. | Ref. | Ref. | Ref. |
| Retired | 0.40 | (0.12,0.68) | 0.32 | (-0.04,0.68) |
| Started work | -0.17 | (-0.6,0.27) | -0.02 | (-0.56,0.51) |
| Continuously retired | 0.29 | (0.11,0.47) | 0.10 | (-0.11,0.31) |
| **Changes in Equivalized household income**  **(10,000 JPY)** | -0.001 | (-0.001,0.0001) | -0.001 | (-0.001,0.0002) |
| **Changes in IADL limitation** | -0.24 | (-0.3,-0.19) | -0.25 | (-0.32,-0.17) |
| **Incidence of stressful life events in the past one year** |  |  |  |  |
| Serious illnesses | 0.58 | (0.3,0.86) | 0.47 | (0.11,0.84) |
| Started family caregiving | 0.37 | (-0.1,0.84) | 0.60 | (0.18,1.02) |
| **Changes in marital status** |  |  |  |  |
| Married – Married | Ref. | Ref. | Ref. | Ref. |
| Married - Not married | 0.34 | (-0.17,0.84) | -0.11 | (-0.51,0.29) |
| Not married – Married | -0.19 | (-1.09,0.7) | 0.54 | (-0.34,1.41) |
| Not married - Not married | 0.01 | (-0.25,0.27) | 0.02 | (-0.16,0.2) |

GDS-15: the short version of the Geriatric Depression Scale (ranging from 0 to 15, higher score indicates more depressive symptoms). Subjects are those who did not show depression at baseline (GDS score < 5). IADL: Instrumental activities of daily living (ranging from 0 to 13, higher score indicates more independency in daily livings). Non-married includes being divorced, widowed, and single.
