# Supplementary material for: Retirement and mental health: dose social participation mitigate the association? A fixed-effects longitudinal analysis
Source: BMC Public Health. 2017 May 30;17:526. doi: 10.1186/s12889-017-4427-0 (PMC5450308; doi:10.1186/s12889-017-4427-0)
Supplement: Supplementary file 3 — Regression Coefficients of Changes in Working Status and Interactions with social participation by gender (sub-sample). (DOCX 14 kb) [file 12889_2017_4427_MOESM3_ESM.docx]

**Table S3.** Regression Coefficients of Changes in Working Status and Interactions with social participation by gender (sub-sample)

| Independent variables | Dependent Variable: Changes in GDS score from 2010 to 2013 | | | | | | | |
| --- | --- | --- | --- | --- | --- | --- | --- | --- |
|  | Model 6 | | | | Model 7 | | | |
|  | Men | | Women | | Men | | Women | |
|  | β coefficient (95%CI) | | | | | | | |
| **Changes in working status (2010 - 2013)** |  |  |  |  |  |  |  |  |
| Kept working | Ref. | Ref. | Ref. | Ref. | Ref. | Ref. | Ref. | Ref. |
| Retired | 0.71 | (0.36,1.06) | 0.56 | (0.04,1.07) | 0.53 | (0.19,0.88) | 0.48 | (-0.03,0.99) |
| Started work | -0.17 | (-0.6,0.27) | -0.02 | (-0.56,0.51) | -0.19 | (-0.61,0.24) | 0.02 | (-0.51,0.55) |
| Continuously retired | 0.29 | (0.11,0.47) | 0.10 | (-0.11,0.31) | 0.22 | (0.05,0.4) | 0.07 | (-0.15,0.28) |
| **Interaction terms** |  |  |  |  |  |  |  |  |
| Retired* No social participation with roles | Ref. | Ref. | Ref. | Ref. | Ref. | Ref. | Ref. | Ref. |
| Retired* Social participation with roles | -0.28 | (-0.9,0.34) | -0.15 | (-0.98,0.68) | -0.27 | (-0.88,0.34) | -0.18 | (-1,0.64) |
| Retired* No recreational social participation | Ref. | Ref. | Ref. | Ref. | Ref. | Ref. | Ref. | Ref. |
| Retired* Recreational social participation | -0.52 | (-1.04,-0.005) | -0.39 | (-1.1,0.33) | -0.35 | (-0.86,0.16) | -0.31 | (-1.02,0.4) |
| **A sense of meaning in life** |  |  |  |  | -1.80 | (-2.12,-1,49) | -1.27 | (-1.55,-0.99) |

Adjusted for changes in time varying confounding factors including equivalised houshold income, IADL limitation, marital status, stressful life events, social relationships. GDS-15: the short version of the Geriatric Depression Scale (ranging from 0 to 15, higher score indicates more depressive symptoms). Subjects are those who did not show depression at baseline (GDS score < 5). Social participation with roles includes volunteering clubs and neighborhood council. Recreational social participation includes sports organizations, hobby clubs, and older adults clubs. A sense of purpose in life: 0=not having, 1=having. Main effects of social participation with roles and recreational social participation were included in the model. Data of sub-sample (only one of the 5 sub-versions of JAGES datasets) was used for these analyses as it is the only version that contains information on a sense of meaning in life
